# Safety of sheathless vascular access using braided 4 F selective catheters for common body interventions – a retrospective study

**DOI:** 10.1186/s42155-023-00350-5

**Published:** 2023-02-16

**Authors:** Jonathan Nadjiri, Tobias Geith, Marc Mühlmann, Tobias Waggershauser, Philipp M. Paprottka

**Affiliations:** Department of Interventional Radiology, School of medicine, Klinikum rechts der Isar, Technical University of Munich, Ismaninger Straße 22, 81675 Munich, DE Germany

**Keywords:** Sheathless, Vascular access, Femoral, Transarterial, Braided catheter, 4 F

## Abstract

**Background:**

Besides other factors, complication rate of transarterial interventions depends on the size of the vascular access. Therefore, the vascular access is mostly chosen as small as possible while still allowing all planned parts of the intervention.

This retrospective analysis is to evaluate the safety and feasibility of sheathless arterial interventions for a broad spectrum of interventions in daily practice.

**Methods:**

All sheathless interventions using a 4 F main catheter between May 2018 and September 2021 were included in the evaluation. Additionally, intervention parameters such as type of catheter, use of microcatheter and required change of main catheters were assessed. Information about the use about sheathless approach and catheters were obtained from the material registration system. All catheters were braided.

**Results:**

503 sheathless interventions with 4 F catheters from the groin were documented. The spectrum comprised bleeding embolization, diagnostic angiographies, arterial DOTA-TATE-therapy, uterine fibroid embolization, transarterial chemotherapy, transarterial radioembolization and others. In 31 cases (6 %) a change of the main catheter was required. In 381 cases (76 %) a microcatheter was utilized. No clinically relevant adverse events were observed (grade 2 or higher [CIRSE AE-classification]). None of the cases later required conversion to a sheath-based intervention.

**Conclusions:**

Sheathless interventions with a 4 F braided catheter from the groin are safe and feasible. It allows for a broad spectrum of interventions in daily practice.

## Background

The main location of adverse events in transarterial interventions is the vascular access (Azzalini et al., 2015; Moran et al., 2001). The complication rate is related to the diameter of the introduced catheter or sheath (Doyle et al., 2008; Kern et al., 1990; Marso et al., 2010; Minici et al., 2020; Moran et al., 2001; Stone & Campbell, 2012). As a result, many interventional centres attempt to use the smallest vascular access that allows for safe conduction of the planned intervention. Several studies have shown reduced complication rates, safety, and feasibility of sheathless interventions at other locations or with small study populations (Mamas et al., 2017; Oguro et al., 2016; Ruzsa et al., 2016). Therefore, this study aimed to evaluate the safety and feasibility of sheathless interventions with braided 4 F catheters from the groin in daily practice.

## Methods

### Acquisition of data

Standard procedures that are not expected to require a change of the mother catheter are usually performed without a sheath. For this study we included all sheathless interventions between May 2018 und September 2021. The information about catheters, intervention characteristics and adverse events were obtained from the hospital information system and the radiology information system.

### Study population and parameters of interest

503 interventions were documented. Parameters of interest were the type of intervention, gender, patient’s age, type of used catheters, use of microcatheters and adverse events. Adverse events were classified according to the CIRSE classification system of adverse events (Filippiadis et al., 2017). Grade 1 events (complication during the procedure which could be solved within the same session; no additional therapy, no post-procedure sequelae, no deviation from the normal post-therapeutic course) were not documented and therefore were not available for analysis. For the choice of the appropriate catheter previous CT scans of the patients were carefully reviewed. CT was not used for evaluation of the access but only for the intervention target.

### Catheters

All selectivecatheters were braided 4 F Tempo® catheters (Cordis®, Cardinal Health, Inc. ®, USA). All used micro catheters were 2.7 F Terumo® (Japan) Progreat® catheter sets with the wire included.

### Description of access

All interventions were conducted without any sedation or analgesia besides local anaesthesia with 5ml scandicain (2%). Arterial puncture was done without image guidance by default. If that approach was unsuccessful fluoroscopy was used or sonographic guidance. Vascular access was gained using a Seldinger needle Cook® (USA) utilizing the double wall technique. For all interventions a 0.035” hydrophilic guide wire was used for vascular access. Neither an incision was made nor were dilators used. Then the main catheter was inserted via the above-mentioned guidewire. In case of a required change of the main catheter, the guidewire was advanced and left in place and the catheter was exchanged analogous to a sheath-based intervention. After the intervention the puncture site was compressed manually for 2-10 min. By default, a pressure bandage for 24h was applied and strict bed rest for 4 hours was recommended.

### Statistics

R Statistics (R version 3.5.3 (2019-03-11) -- "Great Truth") was used for descriptive statistics (Team RC, 2019).

## Results

### Study population

In total, 503 sheathless interventions via an arterial femoral access were analysed. The mean age was 64.8 (± 15.3) and 60% were male; further details are provided in Table 1.

**Table 1:** Patients characteristics

Male	300 (60%)
Age	64.8 (± 15.3)
Indication for angiography	
Bleeding	81 (16%)
Diagnostic angiography	145 (29%)
Abdomen	109 (22%)
Lower extremeties	21 (4%)
Upper extremeties	15 (3%)
Arterial 177-Lu-DOTA-TATE therapy	53 (11%)
Uterus fibroma embolization	41 (8%)
Trans-arterial radioembolization	84 (17%)
Trans-arterial chemotherapy	86 (17%)
Other (e.g. tumor embolization or splenic embolization)	13 (3%)

### Interventions

The indications for interventions are provided in Table 1 and the listing of catheters used is provided in Table 2. Three hundred eighty-one interventions required superselective microcatheters for diagnostic purposes, for embolization including chemoembolization, radioembolization or coiling. In 31 cases (6%), at least one change of the main catheter was necessary due to anatomic circumstances or for interventions/diagnostics of different areas, e.g., lower extremities first and upper extremities second in the same session. During the procedures, the interventionalists noticed improved rotation longitudinal and rotational positioning stability due to the higher friction of the catheter against the surrounding tissue e.g. compared to the low friction of a catheter in a teflon sheath.

**Table 2:** Catheter characteristics

C2-configuration	291 (58%)
RDC1-configuration	102 (20%)
H1- configuration	18 (4%)
Pig-Tail-configuration	3 (< 1%)
S1-configuration	88 (17%)
Other	1 (< 1%)
Use of microcatheter	381 (76%)
Intervention required change of the main selective catheter	31 (6%)

### Adverse events and complications

Peri-interventionally, no issues or complications related to the vascular access occurred. Adverse events were not documented in patients’ medical documents. However, grade 1 adverse events were not specifically evaluated and documented in clinical practice, respectively. No intervention had to be aborted as a consequence of a sheathless approach. No conversion to a sheath-based intervention was required.

## Discussion

The main findings of this study are highly indicative that sheathless access via the groin using braided 4 F catheters is safe and feasible in daily practice for common transarterial interventions.

### Practical aspects

Size of vascular access devices have been proven to be an important factor for patient safety in endovascular medicine (Uhlemann et al., 2012). Although in clinical practice interventionalists attempt to reduce the size of vascular access devices, some studies were not able to detect an effect for smaller sheaths like 4 F vs. 6 F as a cause of major complications; the study did show significantly increased minor complications for 6 F interventions (Chung et al., 2018). The seemingly low number of required catheter changes is a result of two reasons. First, the here described technique has been the standard for one of the authors for a long time. He supervised the included study interventions and advised regarding the catheter choice. Secondly, our study population included a large proportion of liver interventions like trans-arterial chemoembolization or radioembolization. As a consequence, several patients underwent repetitive angiographies. Therefore, the appropriate catheter can be chosen after reviewing the previous angiographies.

#### Advantages

There are several reasons for this approach. Bleeding patients often suffer from impaired coagulation; in those situations, a small puncture lesion seems to be especially worthwhile. Further, the authors have the impression that the required compression time is significantly lower compared to a 4 F sheath which practically has a 6 F outer diameter. Unfortunately, there are no documented compression times available for our study population nor for a comparable matching collective. Therefore, this has to be evaluated in further studies.

The authors did not experience an increased pain perception of the patients compared to sheath-based interventions. Compared to a sheath-based intervention a sheathless approach leads to a better longitudinal and rotational positioning stability of the catheter due to the friction of the catheter with the surrounding tissue. This can be particularly helpful for catheterizing intercostal vessels from top to bottom in search for a bleeding. This characteristic is also very helpful for intraarterial DOTA-TATE treatments. In this situation the catheters remain stable so that patients can be administered the medication in the target artery with relatively high reliability. We also experienced a higher willingness of colleges with limited interventional education to remove just a catheter in comparison to removing a sheath. Furthermore, the positioning stability allows for a self-maintaining rotational preload on a selective catheters which occasionally is required to steer catheter stiffness with torque. Without incisions the vascular access point is practically invisible after 7 days further underlining the minimal invasive character of interventional radiology; this is a subjective observation and was not part of the primary study analysis. However, a certain proportion of the study population underwent repetitive arterial interventions such as trans-arterial radioembolization and the planning angiography shortly before that. The intervals of these interventions usually range from a few days up to two weeks. Trans-arterial chemoembolization often is performed repetitively but with longer intervals. However, after sheathless intervention the pervious puncture site could not be determined after approximately seven days and therefore not be used as orientation for the following interventions. Sheathless interventions can reduce material costs. If a facility only or mainly uses braided catheters, sheathless intervention spares a sheath for every intervention. In most cases the cost of the sheath is half the price of the catheter. In general, selective braided catheters are not significantly more expensive compared to the few non-braided selective catheters on the market. Therefore, sheathless intervention is likely to reduce the material costs even if a facility has to switch from non-braided to braided catheters for the technique.

#### Disadvantages

The main disadvantage remains the slightly different feeling compared to sheath-based interventions; this can be difficult for beginners. The above-mentioned increased positioning stability can be perceived negatively by some interventional radiologists. Especially in hands of less experienced interventionalists there is risk of a subcutaneous malrotation of the catheter and subsequent tear off in the worst case. Insertion of the catheter via a guide wire without a sheath and without an incision is associated with high resistance; especially the skin and the arterial wall cause punctually high resistance. However, after insertion of the tip of the catheter into the arteria the resistance is neglectable. Additionally, a used and warmed catheter might not be stiff enough to penetrate skin and the arterial wall via a guide wire.

It is to assume that repeated change of the main catheter without a sheath causes increased trauma to the arterial wall. Therefore, in the authors’ daily practice sheath-less angiography is only attempted after careful evaluation of previous angiographies or CT-scans. Only in case of a very high likelihood that successful intervention is possible with just one catheter, a sheathless approach was attempted. However, in 6 % of the cases in this study at least one exchange of the main catheter was performed without a sheath. Even in these cases no relevant adverse events occurred indicating that exchange of catheters without a sheath might be safe.

Sheathless access was not attempted with non-braided catheters; it is to assume that it might be more difficult if not impossible but has to be supported by data.

### Limitations

This is a single centre study. Adverse events of the category 1 were not documented; even though occurrence of category 1 adverse events is clinically irrelevant this study could not evaluate the incidence of category 1 events with 4 F sheathless interventions from the groin. This study did not compare the sheathless approach against sheath-based interventions in a randomized study nor were compression times documented that could be compared. No relevant adverse events were observed in the entire study, therefore the precise complication rate cannot be calculated. The number of atherosclerotic patients was not determined.

## Conclusion

Sheathless vascular access with braided 4 F catheters is safe and feasible for common body interventions in daily practice. Sheathless interventions might especially be interesting for facilities performing a large number of transarterial chemoembolization and/or radioembolization including angiographies for radioembolization planning.

## Data Availability

The data file is available on reasonable request from the corresponding author.
